# Identifying Patterns of Smoking Cessation App Feature Use That Predict Successful Quitting: Secondary Analysis of Experimental Data Leveraging Machine Learning

**DOI:** 10.2196/51756

**Published:** 2024-05-22

**Authors:** Leeann Nicole Siegel, Kara P Wiseman, Alex Budenz, Yvonne Prutzman

**Affiliations:** 1 National Cancer Instiute National Institutes of Health Rockville, MD United States; 2 University of Virginia School of Medicine Charlottesville, VA United States

**Keywords:** smartphone apps, machine learning, artificial intelligence, smoking cessation, mHealth, mobile health, app, apps, applications, application feature, features, smoking, smoke, smoker, smokers, cessation, quit, quitting, algorithm, algorithms, mobile phone

## Abstract

**Background:**

Leveraging free smartphone apps can help expand the availability and use of evidence-based smoking cessation interventions. However, there is a need for additional research investigating how the use of different features within such apps impacts their effectiveness.

**Objective:**

We used observational data collected from an experiment of a publicly available smoking cessation app to develop supervised machine learning (SML) algorithms intended to distinguish the app features that promote successful smoking cessation. We then assessed the extent to which patterns of app feature use accounted for variance in cessation that could not be explained by other known predictors of cessation (eg, tobacco use behaviors).

**Methods:**

Data came from an experiment (ClinicalTrials.gov NCT04623736) testing the impacts of incentivizing ecological momentary assessments within the National Cancer Institute’s quitSTART app. Participants’ (N=133) app activity, including every action they took within the app and its corresponding time stamp, was recorded. Demographic and baseline tobacco use characteristics were measured at the start of the experiment, and short-term smoking cessation (7-day point prevalence abstinence) was measured at 4 weeks after baseline. Logistic regression SML modeling was used to estimate participants’ probability of cessation from 28 variables reflecting participants’ use of different app features, assigned experimental conditions, and phone type (iPhone [Apple Inc] or Android [Google]). The SML model was first fit in a training set (n=100) and then its accuracy was assessed in a held-aside test set (n=33). Within the test set, a likelihood ratio test (n=30) assessed whether adding individuals’ SML-predicted probabilities of cessation to a logistic regression model that included demographic and tobacco use (eg, polyuse) variables explained additional variance in 4-week cessation.

**Results:**

The SML model’s sensitivity (0.67) and specificity (0.67) in the held-aside test set indicated that individuals’ patterns of using different app features predicted cessation with reasonable accuracy. The likelihood ratio test showed that the logistic regression, which included the SML model–predicted probabilities, was statistically equivalent to the model that only included the demographic and tobacco use variables (*P*=.16).

**Conclusions:**

Harnessing user data through SML could help determine the features of smoking cessation apps that are most useful. This methodological approach could be applied in future research focusing on smoking cessation app features to inform the development and improvement of smoking cessation apps.

**Trial Registration:**

ClinicalTrials.gov NCT04623736; https://clinicaltrials.gov/study/NCT04623736

## Introduction

Cigarette smoking remains a leading cause of preventable death in the United States [1]. Evidence-based smoking cessation interventions, though proven to be valuable in helping people quit, are underused [2]. Smartphone apps have the potential to expand the reach and increase the use of evidence-based smoking cessation interventions [1,3]. Smartphone ownership is high among every demographic group in the United States [4], and an array of smoking cessation apps, including many free options [5], are available in smartphone app stores. Evidence suggests that smoking cessation apps are widely used, with millions of downloads per year [6,7].

Research supporting the use of apps for smoking cessation is still emerging, and many publicly available apps have not been rigorously tested [8]. However, results from randomized controlled trials (RCTs) suggest that apps can be effective in helping people quit smoking [9-11]. Studies have also demonstrated that both higher user engagement in smoking cessation apps [11,12] and longer duration and greater frequency of app use [6] are related to smoking cessation.

The many capabilities, features, and functionalities that can be incorporated into smoking cessation apps have the potential to increase their effectiveness. Apps can include interactive and multimedia content, and offer tailored features to meet the needs and preferences of different types of users [13]. Several reviews have cataloged the most common types of features in smoking cessation apps and evaluated whether those features align with behavioral theories or smoking cessation clinical guidelines [5,14-17]. Some studies have also investigated whether and how users respond to and use particular app features. Through their content analysis of smoking cessation app reviews and ratings, Bendotti et al [18] found that users liked app features that allowed them to set goals, track their progress, understand and manage their cigarette cravings, and interact with others within the app. Hoeppner et al [13] found that apps using tailored communications with users were more likely to have received more than 10,000 downloads compared to apps that did not use tailored communications. In a recent study focused on the National Cancer Institute’s quitSTART app, the app used in this study, Budenz et al [19] found that a substantial proportion of users accessed app-integrated, mood-related support.

Few studies have examined the impacts of using particular app features on smoking cessation outcomes. Rajani et al [20] found that increased frequency of use of their apps’ gamification features (eg, earning badges and unlocking levels) was associated with increases in perceived self-efficacy and motivation to quit smoking. Heffner et al [21] looked at features within a smoking cessation app that was both popular (ie, among the 10 most-used features in the app) and significantly associated with successful quitting and identified 2 app features that met both criteria—viewing one’s quit plan and tracking one’s practice of letting smoking urges pass. In their study focused on a smoking cessation app that emphasized positive psychology content, Hoeppner et al [22] found that greater engagement with the app’s happiness-related features was predictive of cessation.

More research is needed to understand which smoking cessation app features are most valuable in helping users quit smoking. Fortunately, the apps are designed to efficiently collect user data that can be used to answer this question. App developers can record users’ activity within apps, capturing information such as how many times and when an individual took an action within the app and how quickly they responded to an app notification. However, raw app user data can be large and unwieldy, particularly for apps that offer many features and garner frequent engagement from users. Machine learning methods expand our ability to analyze and glean insights from app user data. The use of machine learning methods to analyze user data from smoking cessation apps has the potential to optimize the effectiveness of such apps [23,24].

In this study, we leverage supervised machine learning (SML) methods to conduct a secondary analysis of app user data collected from participants as part of an RCT involving the quitSTART smoking cessation app—the quitSTARTEMA Incentivization Trial. Our primary goal in conducting this study is to outline an analytic approach that could be used in future studies investigating whether and how patterns of use of different smoking cessation app features affect cessation. We also seek to fulfill the following exploratory research aims: (1) examine the extent to which patterns of use of different features of the quitSTART app can be used to predict participants’ short-term smoking cessation and (2) test whether participants’ patterns of app feature use predict variance in short-term cessation that is not predicted by other variables related to smoking cessation.

## Methods

### The quitSTART App

The quitSTART app is a free, publicly available app created by the National Cancer Institute’s Smokefree.gov initiative, a federal program that offers no-cost, evidence-based tobacco cessation support to the public through a suite of websites, text messaging programs, and mobile apps [25]. The quitSTART app is available for both iPhones and Androids and is popular, with 10,000-20,000 new downloads each year [25].

The app offers a range of features designed to assist individuals in quitting smoking. App users can explore content pages, referred to as “cards,” which contain information, tips, and inspiration for quitting smoking. They can also seek real-time support for managing their cravings, mood, and handling slips; play games to distract themselves during cravings; track their progress; and earn badges as they continue to use the app. Users can customize their app experience by building a “quit kit” containing cards they find useful and can create custom notifications. Since 2017, the quitSTART app has also included ecological momentary assessment (EMA) capability. By default, users are sent 3 EMA prompts each day at random times to report their craving level, mood, and number of cigarettes smoked. Users can opt out of receiving EMAs by disabling notifications from the app.

### Experimental Design

Data for this analysis were drawn from an experimental trial conducted between October 2020 and May 2021. The quitSTART EMA Incentivization Trial was conducted to test the effects of incentivizing EMA completion within the quitSTART app on short-term smoking cessation. Participants were English-speaking adults who lived in the United States, smoked cigarettes, and had a self-reported desire to quit smoking.

As the goal of the clinical trial was to test the effects of incentivizing completion of EMAs on smoking cessation, eligible participants were randomized 1:1 into 2 study arms, an incentivized EMA arm and a nonincentivized EMA arm. Participants randomized to the nonincentivized EMA arm were compensated for completing the surveys administered to all participants at baseline, 2 weeks into the study, and at the end of the 4-week study. Participants in the nonincentivized arm received EMA notifications, which are sent to all users by default. However, their compensation was not affected by their EMA completion. In contrast, participants randomized to the incentivized EMA arm were informed that part of their compensation would be contingent on completing surveys and the other part would be contingent on their EMA participation. They had to complete at least half of the programmed EMAs to receive any EMA compensation, and increasing EMA participation resulted in higher compensation. The total amount of compensation that could be earned was identical across the 2 study arms.

After completing the baseline survey, participants were instructed to download the quitSTART app and use it for the 4-week study period. A total of 152 participants completed the enrollment process and participated in the study, of whom 133 (88.2%) completed the 4-week follow-up survey. These 133 participants were included in this study. Figure S1 in Multimedia Appendix 1 summarizes the recruitment, randomization, and data collection processes for this study.

### Ethical Considerations

The University of Virginia institutional review board approved the study design and protocol (UVA SBS IRB protocol 3643; ClinicalTrials.gov NCT04623736).

### Study Measures

#### Baseline Participant Characteristics

Data collected in the baseline survey included participants’ gender identity, sexual orientation, education level, and scores on the Patient Health Questionnaire-9 (PHQ-9) [26], which is used to measure the presence and severity of depressive symptoms. The baseline survey also assessed participants’ use of tobacco products, nicotine dependence scores [27], and whether they had made an attempt to quit within the past year. When participants downloaded and first used the app, their phone type (ie, whether they had an Android or iPhone) was recorded.

#### Smoking Cessation Outcome Measure

The outcome of interest for this study, short-term cigarette smoking cessation, was measured at the end of the quitSTART EMA Incentivization Trial and was operationalized as 7-day point-prevalence abstinence at 4 weeks postenrollment. Participants were asked, “Have you smoked a cigarette (even a puff) in the past seven days?” Participants who responded “no” to this question were considered to have quit smoking.

#### App Feature Use Variables

As participants used the quitSTART app, each action they took and its corresponding time stamp were recorded. These data were used to create 3 sets of variables reflecting the participants’ use of app features. The first set of variables, “binary app feature use variables,” consisted of yes or no variables that reflected whether a participant took the action in question; these variables were used for actions that most participants took only 1 time (eg, completing the initial profile set-up process).

For actions that were intended to be taken as many times as a participant wanted (eg, playing a game), 2 additional sets of variables were created. One set of variables used in our main analyses, which we labeled “proportion app feature use variables,” reflected the number of times a participant took a particular action within the app divided by their total number of app use sessions. An app use session was defined as a period during which a participant performed 1 or more actions in the app with no more than 2 minutes between actions. We took this approach to ensure that we captured variation in how participants spent their time within the app rather than just variation in the total time they spent in the app. The other set of variables, “count app feature use variables,” reflected the total number of times participants took an action and were used in a sensitivity analysis, as described below.

### Data Analysis

#### Overview

All analyses were conducted in R (version 4.1; R Core Team). We first examined descriptive statistics for the baseline participant characteristics. We also examined participants’ responses to our short-term smoking cessation item.

Our machine learning approach was based on the recommendations made by Dinga et al [28] for controlling for the effects of confounding variables on machine learning predictions. Dinga et al [28] argued that regressing out confounding variables from each predictor variable separately prior to conducting machine learning modeling is insufficient. They instead proposed controlling for confounding variables post hoc at the level of machine learning predictions. We adopted this approach for 2 reasons. First, it allowed us to control for confounding more efficiently. It also enabled us to fulfill our second study aim by testing whether predictions from our machine learning model, which included input variables capturing participants’ use of different app features, explained variance in cessation that was not explained by participant-level variables that could potentially affect cessation such as demographic characteristics and tobacco use.

#### Aim 1 Analysis

To identify which patterns of use of app feature use predict short-term smoking cessation, we built SML models predicting 7-day smoking abstinence from a set of predictor variables that included our binary app feature use variables, our proportion app feature use variables, participants’ total number of app use sessions, phone type (iPhone or Android), and study arm. Phone type was included as a variable in the SML models because the iPhone and Android versions of the quitSTART app were built separately and user data from each app were recorded in a slightly different manner. Although the 2 apps appeared identical to users and we harmonized the user data collected from each, we chose to include phone type as a variable in the SML models in case there was a relationship between phone type and app use or between phone type and cessation. We first randomly divided our data into a training set (n=100, 75% of the data) and a held-aside test set (n=33, 25% of the data).

Working with the training set, we used recursive feature elimination with 10-fold cross-validation to determine the optimal number of features for our classifier and then fit our logistic regression classifier using this number of features to the training set. We selected a logistic regression classifier because our outcome variable was binary, and we wanted a classifier that would yield predicted probabilities (rather than binary predictions) for every participant. We evaluated the SML model’s performance in the training set by looking at its sensitivity, specificity, and accuracy. We also examined variable importance (defined as the scaled absolute value of the coefficient of each variable in a logistic regression model for binary classification) for each feature and identified the features in the model assigned the highest importance for predicting cessation. We produced partial dependence plots for each of the top 10 most important features in order to better understand each feature’s relationship with short-term smoking cessation [29].

We then applied the model to the held-aside test set and looked at its sensitivity, specificity, and accuracy. We then used it to produce predicted probabilities of short-term cessation for each participant included in the test set.

#### Aim 2 Analysis

As a first step toward testing whether participants’ patterns of app feature use predicted unique variance in cessation, we fit 2 logistic regression models using the test set data. These models were fit with all participants in the test set who were not missing data on any demographic or participant characteristic variables (n=30; a total of 3 participants were excluded from the aim 2 analyses because of missing data on the gender variable). Due to the small sample size available, no data splitting or cross-validation was performed. The first model included participant demographic variables, as well as other variables that prior research suggests may be related to cessation. These variables were measured in the baseline survey and included age, race or ethnicity, gender identity, education, PHQ-9 scores, sexual orientation, nicotine dependence, quit attempts in the past year, and polytobacco use. The second model included all these variables, as well as an additional predictor variable—the predicted probabilities of short-term cessation from the SML model. After fitting each model, we assessed its fit through a likelihood ratio test comparing it to a null model. We then ran a likelihood ratio test comparing the 2 logistic regression models to one another to assess whether the model that included the SML model-predicted probabilities of cessation had a significantly better fit to the data.

#### Sensitivity Analysis

As a sensitivity analysis, we repeated our aim 1 and aim 2 analyses with 1 major change. We used the count app feature use variables in place of the proportion app feature use variables in our SML model. Participants’ total number of app use sessions was not included as a predictor in these models due to its collinearity with the count app feature use variables.

## Results

Descriptive statistics for participant characteristics measured in the baseline survey, as well as participants’ study arm and phone type, are summarized in Table 1. Descriptive statistics are shown for all participants, as well as for participants who were included in the training set (n=100) and in the test set (n=33) when building our SML models. Among all 133 participants in the study, 62 (46.6%) were randomized to the incentivized EMA arm. About half (n=74, 55.6%) of participants had iPhones, while 59 (44.4%) had Androids. Participants’ average age was 45.6 (SD 12.6) years. Participants reported being mostly non-Hispanic White (n=103, 77.4%), female (n=99, 74.4%), and straight (n=106, 79.7%). The average PHQ-9 score was 7.8 (SD 6.1), which indicates mild depression [26].

Participants’ mean score on the Fagerstrom test was 4.8 (SD 2.4), which equates to medium nicotine dependence [30]. Most participants (n=105, 78.9%) had made a prior attempt to quit smoking within the past year. Approximately a third of participants (n=46, 34.6%) reported polytobacco use. Roughly a quarter (n=37, 27.8%) of participants reported 7-day point-prevalence abstinence at 4 weeks.

The full list of variables that were considered for inclusion in the SML model and their descriptions are included in Table 2. Results from recursive feature elimination showed that 28 features out of 29 candidate features should be included in the SML model (every feature except ncravingspressed_prop). We ran our SML model including these 28 features in the training set and assessed its performance. The model’s accuracy in the training set was 0.91, its sensitivity was 0.96, and its specificity was 0.79.

**Table 1:** Baseline participant characteristics for all participants, training set, and test set.

Characteristics			All participants (N=133)		Training set (n=100)		Test set (n=33)
**Study arm, n (%)**							
	Incentivized EMA^a^ arm	62 (46.6)		45 (45)		17 (51.5)	
	Nonincentivized EMA arm	71 (53.4)		55 (55)		16 (48.5)	
**Phone type, n (%)**							
	iPhone	74 (55.6)		53 (53)		21 (63.6)	
	Android	59 (44.4)		47 (47)		12 (36.4)	
Age (years), mean (SD)			45.6 (12.6)		47.0 (12.4)		41.5 (12.3)
**Race or ethnicity, n (%)**							
	Non-Hispanic White	103 (77.4)		77 (77)		26 (78.8)	
	Hispanic White	30 (22.6)		23 (23)		7 (21.2)	
**Sex, n (%)**							
	Male	31 (23.3)		25 (25)		6 (18.2)	
	Female	99 (74.4)		75 (75)		24 (77.4)	
	Missing	3 (2.3)		0 (0)		3 (9.1)	
**Education level, n (%)**							
	Less than high school	6 (4.5)		5 (5)		1 (3)	
	High school graduate or equivalent	11 (8.3)		8 (8)		3 (9.1)	
	Some college	50 (37.6)		37 (37)		13 (39.4)	
	College graduate or more	66 (49.6)		50 (50)		16 (48.5)	
**Sexual minority status, (%)**							
	Straight	106 (79.7)		84 (84)		22 (66.7)	
	Not straight	27 (20.3)		16 (16)		11 (33.3)	
PHQ-9^b^ score, mean (SD)			7.8 (6.1)		8.07 (6.32)		7.15 (5.59)
Fagerstrom test, mean (SD)			4.8 (2.4)		4.56 (2.31)		5.52 (2.55)
**Quit attempt in past 12 months, n (%)**							
	Yes	105 (78.9)		78 (78)		27 (81.8)	
	No	28 (21.1)		22 (22)		6 (18.2)	
**Poly-use of tobacco products, n (%)**							
	Yes	46 (34.6)		34 (34)		12 (36.4)	
	No	87 (65.4)		66 (66)		21 (63.6)	

^a^EMA: ecological momentary assessment.

^b^PHQ-9: Patient Health Questionnaire-9.

**Table 2:** Variables considered for inclusion in SML^a^ model (n=29), definitions, and mean values among participants (N=133).

Variable name			Definition		Values
**Proportion app feature use variables (n=24), mean (SD)**					
	naddlocation_prop	How many times a participant added a location to receive a location-based notification app use divided by their app use sessions.		0.02 (0.06)	
	naddtime_prop	How many times a participant selected a specific time of day for a time-based notification divided by their app use sessions.		0.03 (0.09)	
	nbadgescompleted_prop	How many badges a participant earned for reaching milestones in their app use or cessation journey divided by their app use sessions.		0.55 (0.45)	
	nbadgesviewed_prop	How many times a participant viewed a badge available to earn divided by their app use sessions.		0.01 (0.03)	
	nbuttonsfavorited_prop	How many times a participant favorited a content page divided by their app use sessions.		0.74 (2.44)	
	nbuttonsshared_prop	How many times a participant shared a content page divided by their app use sessions.		0.03 (0.08)	
	ncardsviewed_prop	How many content pages a participant viewed divided by their app use sessions.		4.61 (4.33)	
	nchallengesaccepted_prop	How many times participants accepted a challenge divided by their app use sessions.		0.05 (0.07)	
	ncompletedemas_prop	How many EMA^b^ prompts a participant completed divided by their app use sessions.		0.18 (0.17)	
	ncravingspressed_prop	How many times a participant pressed the “I’m Craving” button divided by their app use sessions.		0.07 (0.09)	
	ncustomtips_location_prop	How many times a participant entered a custom notification to receive at a specific location divided by their app use sessions.		0.00 (0.02)	
	ncustomtips_time_prop	How many times a participant entered a custom notification to receive at a specific time of day divided by their app use sessions.		0.01 (0.02)	
	nexplorecontentpages_prop	How many times a participant viewed “Tips,” “FYIs” or “Inspirations” content pages divided by their app use sessions.		1.24 (1.08)	
	nfeelingdownpressed_prop	How many times a participant selected the “Feeling Down” button divided by their app use sessions.		0.04 (0.06)	
	nfeelinggreatpressed_prop	How many times a participant selected the “I’m Great” button divided by their app use sessions.		0.10 (0.14)	
	nlocationtags_prop	How times a participant tagged a specific location divided by their app use sessions.		0.00 (0.02)	
	nnotificationsreceived_prop	How many times a participant opened a scheduled notification from the app divided by their app use sessions.		0.64 (0.31)	
	nprogresspressed_prop	How many times a participant pressed the “Progress” button to view their progress in their cessation journey divided by their app use sessions.		0.38 (0.35)	
	nquitdateset_prop	How many times participants set a new quit date divided by their app use sessions.		0.14 (0.24)	
	nregistrations_prop	How many times a participant registered their account divided by their app use sessions.		1.18 (1.21)	
	nscreensviewed_prop	How many screens a participant viewed in the app divided by their app use sessions.		7.52 (3.23)	
	nslippedpressed_prop	How many times a participant selected the “I Slipped” button divided by their app use sessions.		0.10 (0.13)	
	ntimetags_prop	How many times a participant tagged a specific time divided by their app use sessions.		0.00 (0.01)	
	ntotalgames _prop	How many times a participant played a game divided by their number of app use sessions.		0.10 (0.19)	
**Binary app feature use variables (n=2), n (%)**					
	noquitdate_bin	Did a participant opt not to select a quit date while setting up their profile?		23 (17.3)	
	quitdatereset_bin	Did a participant reset their quit date at least once?		47 (35.3)	
**Other variables (n=3)**					
	nunique_sessions, mean (SD)	A participant’s total number of app use sessions, defined as any series of actions within the app with no more than 2 minutes between actions.		54.58 (67.58)	
	Phonetype, n (%)	Did a participant have an iPhone?		74 (55.6)	
	Studyarm, n (%)	Was the participant assigned to the incentivized EMA or the nonincentivized EMA arm?		62 (46.6)	

^a^SML: supervised machine learning.

^b^EMA: ecological momentary assessment.

**Figure 1:**
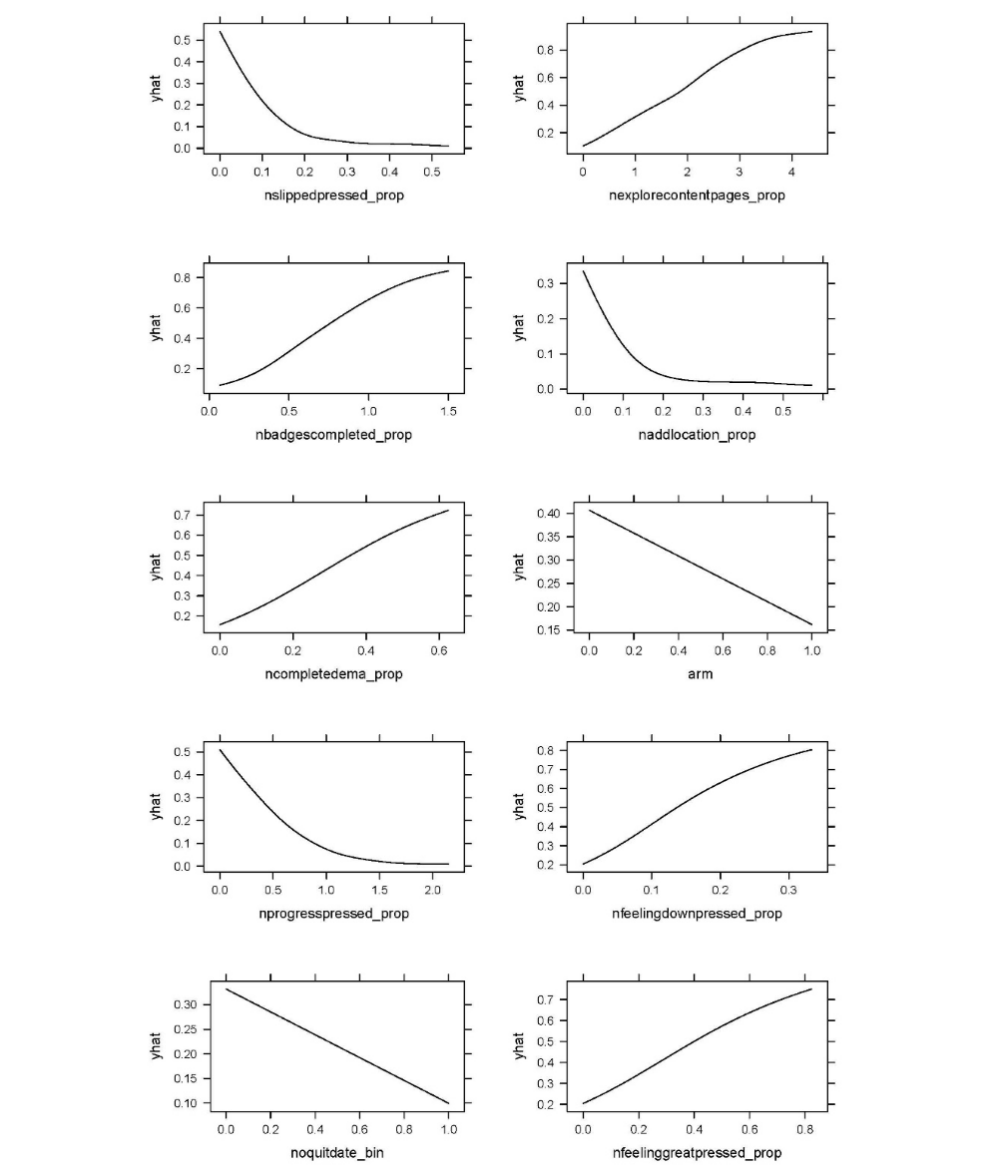
Partial dependence plots depicting the predicted marginal effects on the probability of cessation for the 10 app use variables assigned the highest feature importance. The x-axis in each figure is constrained to show only values of each variable that were observed in the training set used to build the supervised machine learning model.

The importance metrics for all 28 features in the model are displayed in Figure S2 in Multimedia Appendix 1. Partial dependence plots for the 10 most important features are shown in Figure 1. These plots depict the marginal effect of each feature on the probability of smoking cessation. The feature in the model with the highest variable importance was nslippedpressed_prop, the number of times a participant pressed the “I slipped” button divided by their number of app use sessions. By pressing this button, users access targeted content and guidance intended to help them after they had “slipped up” and smoked a cigarette. As can be seen in Figure 1, this feature was negatively related to the probability of cessation, indicating that users who reported “slipping up” more often, proportional to their app use, were less likely to successfully quit smoking. The second and third most important features in the model, respectively, were nexplorecontentpages_prop and nbadgescompleted_prop. The former variable represents the number of times a participant viewed “Tips,” “FYIs,” or “Inspirations” content pages in the app divided by their number of app use sessions. The latter represents the number of badges a participant earned for reaching milestones in their cessation journey or use of the quitSTART app (eg, checking the app 5 times in 1 day) divided by their number of app use sessions. Both of these variables were positively related to cessation, showing that participants who used these app features more often were more likely to successfully quit smoking. Other features that were among the top 10 with the highest variable importance were naddlocation_prop, ncompletedemas_prop, studyarm, nprogresspressed_prop, noquitdate_bin, and nfeelinggreatpressed_prop.

After building our SML model and assessing feature importance in the training set, we fit the model in the test set. The model’s accuracy was 0.67, and both its sensitivity and specificity were also 0.67. We retained the SML model–predicted probabilities of cessation as a variable in the test set.

Results from the 2 logistic regression models performed in the test set are summarized in Table S1 in Multimedia Appendix 1. The likelihood ratio test comparing model 1, which included our set of participant characteristics that are known to be related to cessation, to a null model was not statistically significant at the α=.05 level (*χ*^2^_9_=5.0; *P*=.84). Likewise, the likelihood ratio test comparing model 2, which included all variables included in model 1, as well as the predicted probabilities of cessation from the SML model to a null model was not statistically significant (*χ*^2^_10_=7.0; *P*=.73). The likelihood ratio test comparing model 2 to model 1 was not statistically significant (*χ*^2^_1_=2.0; *P*=.16), indicating that model 2 provided a statistically equivalent fit to the data to model 1.

The variables considered for inclusion in our sensitivity analysis SML model are summarized in Table S2 in Multimedia Appendix 1. Recursive feature elimination showed that the optimal number of features to include in the model was 28. The model’s accuracy in the training set was 0.88, its sensitivity was 0.94, and its specificity was 0.71. The most important feature of the model was studyarm, which represented the participants’ assigned study arm. The importance metrics for each feature included in the model are displayed in Figure S3 in Multimedia Appendix 1 and each feature is defined in Table S2 in Multimedia Appendix 1. The model’s accuracy in the test set was 0.64. Its sensitivity was 0.75 while its specificity was 0.33.

We fit 2 logistic regression models in the test set (see Table S3 in Multimedia Appendix 1) and ran a likelihood ratio test comparing the 2 models. The likelihood ratio test was not statistically significant (*χ*^2^_1_=0.6; *P*=.46), indicating that model 2, which included the predicted probabilities from the SML model using continuous app feature use variables, did not provide a significantly better fit to the data than model 1.

## Discussion

### Principal Findings

We developed and tested a novel approach to using SML to examine whether and how the use of specific features within a smoking cessation app predicts short-term cessation. We applied SML models to data from the quitSTART EMA Incentivization Trial to identify patterns of app feature use that predict short-term smoking cessation. Our analysis of variable importance within this model indicated that the 3 app feature use variables that were most important for predicting cessation were the number of times participants pressed the “I Slipped” button, the number of times they viewed the “Tips,” “FYIs,” or “Inspirations” content pages, and the number of badges they completed (each expressed as a proportion of total app use sessions). We then used a likelihood ratio test comparing 2 logistic regression models to assess whether including patterns of app feature use in our models allowed us to better predict cessation. The results of this likelihood ratio test showed that the logistic regression model that included both the SML-predicted probabilities of cessation based on participants’ app feature use, as well as a set of variables reflecting participants’ baseline tobacco use and demographic and personal characteristics did not fit the data better than a model that included only the latter variables. This means the accuracy of our model predicting whether participants quit smoking was not improved by including the SML-predicted probabilities. However, because only observations from the held-aside test set (n=30) were included in this analysis, the small n likely contributed to this null result.

This study adds to the small but growing body of literature that has gone beyond looking at the overall relationship between smoking cessation app use and smoking cessation to examine which specific app features are associated with cessation [20,21]. Some of our findings align with those from prior research. For example, our finding that completing badges is an important variable for predicting smoking cessation aligns with the finding reported by Rajani et al [20] that participants’ frequency of use of gamification features, including earning badges, was associated with motivation to quit. However, there is a need for more research investigating different app features within smoking cessation apps to help maximize the potential public health impacts of smoking cessation apps. The methodological approach developed in this study could be used to guide additional research evaluating smoking cessation apps and to improve the design and refinement of such apps. While this study focused on smoking cessation, this approach could also be applied in research on apps focused on other health behaviors.

Our methodological approach could help guide further research in several ways. For example, our finding that patterns of app feature use did not predict unique variance in cessation might lead researchers to explore whether there is variability in the extent to which different groups of app users are helped by different app features. Alternatively, finding that patterns of app feature use did predict unique variance in cessation might inspire additional research investigating users’ perceptions of, satisfaction with, and reasons for using the app feature use variables that were found to be important for predicting cessation.

Additionally, if an app feature uses a variable that was expected to be effective based on theory and prior research was not found to be important in predicting cessation, researchers might investigate why this was the case, considering possible explanatory factors such as design and usability issues [15,18]. This research could also inform the design of new apps, as well as the refinement of existing apps. Apps could be streamlined to only include features found to be important for cessation, which could in turn improve their cost-efficiency for app developers and usability for app users.

While this was a retrospective analysis conducted after participants had finished using quitSTART, SML models could also be applied in real time to identify current app users whose patterns of app feature use suggest they may be unlikely to quit smoking. These individuals could then be sent tailored messages through the app to nudge them to alter their patterns of app use or connect them with additional support. For example, in this study, we found that individuals who pressed the “I slipped” button more frequently, proportional to their overall app use, were less likely to report short-term smoking cessation. If this relationship was observed in a context in which real-time intervention was possible, individuals who pressed the “I slipped” button could automatically be connected to another source of support, such as a smoking cessation counselor.

### Study Limitations

Given that this was a secondary data analysis involving a relatively small convenience sample of individuals who participated in an experiment, findings from this study were not expected to be generalizable to the general population of people who smoke. Findings were also not expected to be generalizable to all quitSTART users because the experimental protocol itself may have affected some participants’ app feature use. Specifically, participants in the incentivized EMA arm received compensation based on their completion of EMAs and, as a result, used that app feature more frequently than did participants in the nonincentivized EMA arm (unpublished data, 2021). The small sample size, as well as the relative rarity of our cessation outcome (about 28% of participants reported 7-day point-prevalence abstinence at 4 weeks), may also have impacted the accuracy of the SML model we fit, contributing to its suboptimal accuracy, specificity, and sensitivity in the test set. These factors may also have affected the results of our aim 2 statistical analyses.

Additionally, the app feature use variables we included in our SML model only captured the number of times a participant used a given app feature as a proportion of their overall app use or whether the participant had used an app feature at all. Future research should examine factors such as the time of day during which a participant used a given app feature or the responses given to interactive app features to get a more detailed view of the relationship between app feature use and cessation. Finally, although we accounted for several variables that might be related to cessation in our logistic regression models, the list of variables we included was not exhaustive.

### Conclusions

Smartphone apps could expand the availability and use of evidence-based smoking cessation interventions, potentially helping more people quit smoking. However, there is a need for more research evaluating the effectiveness of smoking cessation apps and investigating how individuals’ use of different app features impacts their likelihood of cessation. In this study, we developed and tested a novel methodological paradigm using SML to test patterns of app feature use that are most predictive of short-term smoking cessation and assess whether patterns of app feature use explain variance in cessation that is not explained by other relevant variables. We identified important app feature use variables for predicting cessation. We did not find evidence that patterns of app feature use explained variance in cessation beyond what was explained by participants’ tobacco use and demographic and personal characteristics, although the small sample size likely contributed to this result. Nonetheless, the methodological approach developed in this study could be used in future research focused on smoking cessation apps and health behavior apps more broadly to inform the design and refinement of such apps.

## Appendix

Multimedia Appendix 1 Full results from logistic regression models; results and description of sensitivity analyses; and details about participant recruitment, sample allocation, and data collection.

## Abbreviations

EMA: ecological momentary assessment
PHQ-9: Patient Health Questionnaire-9
RCT: randomized controlled trial
SML: supervised machine learning
