# Supplementary material for: Identifying Patterns of Smoking Cessation App Feature Use That Predict Successful Quitting: Secondary Analysis of Experimental Data Leveraging Machine Learning
Source: JMIR AI. 2024 May 22;3:e51756. doi: 10.2196/51756 (PMC11153975; doi:10.2196/51756)
Supplement: Multimedia Appendix 1 [file ai_v3i1e51756_app1.docx]

**Table S1.** Logistic regression models predicting short-term cessation from participant characteristics only (model 1) or participant characteristics and SML-predicted probabilities of cessation (model 2).

|  | | *Model 1 (n=30)* | | *Model 2 (n=30)* | |
| --- | --- | --- | --- | --- | --- |
| Variable | | Odds Ratio^1^ | 95% CI | Odds Ratio | 95% CI |
| (Intercept) | | 1.73 | [0.00, 1920.03] | 2.86 | [0.00, 7650.59] |
| White Non-Hispanic | | 0.26 | [0.02, 3.00] | 0.13 | [0.01, 2.30] |
| Female | | 0.92 | [0.07, 12.54] | 0.92 | [0.06, 13.60 |
| Education | |  |  |  |  |
|  | More than high school degree | 0.68 | [0.03, 14.04] | 0.52 | [0.02, 14.31] |
| PHQ-9 score | | 1.01 | [.85, 1.19] | 1.05 | [0.87, 1.27] |
| Sexual minority status | | 0.30 | [0.03, 3.23] | 0.48 | [0.04, 6.44] |
| Age | | 1.01 | [0.93, 1.10] | 0.99 | [0.90, 1.10] |
| Fagerstrom Score | | 0.82 | [0.51, 1.33] | 0.85 | [0.51, 1.40] |
| Quit attempt in past year | | 1.98 | [0.10, 19.62] | 1.27 | [0.04, 41.07] |
| Polyuse | | 2.42 | [0.30, 19.62] | 1.90 | [0.22, 16.69] |
| SML-predicted probabilities | |  |  | 6.50 | [0.40, 106.80] |
| Observations (N) | | 30 | | 30 | |
| Pseudo R^2^ | | 0.14 | | 0.19 | |
| **p*< .05; ***p*<.01 | | | | | |
| 1. *Note*. For all binary predictor variables, the odds ratio represents the ratio of the odds of an individual reporting short-term cessation if they have the listed value of the variable (e.g., Female) and the odds of an individual reporting short-term cessation if they do not have the listed value of the variable. For the continuous predictor variables, the odds ratio represents the increase or decrease in the odds of short-term cessation associated with a one-unit change in the predictor variable. | | | | | |

| **Table S2.** Descriptions of variables entered into recursive feature elimination to determine ideal set of variables to include in sensitivity analysis SML model (n=29). | |
| --- | --- |
| ***App Feature Use Variables*** | |
| *Count App Feature Use Variables (n=25)* | |
| naddlocation | The total number of times a participant entered a location at which to receive a location-based notification. |
| naddtime | The number of times a participant selected a specific time of day for a time-based notification. |
| nbadgescompleted | The number of badges a participant earned for reaching milestones in their app use or cessation journey. |
| nbadgesviewed | The number of times a participant viewed a badge available to earn. |
| nbuttonsfavorited | The number of times a participant favorited a content page. |
| nbuttonsshared | The number of times a participant shared a content page. |
| ncardsviewed | The number of content pages a participant viewed divided. |
| nchallengesaccepted | The number of times participants accepted a challenge. |
| ncompletedemas | The number of ecological momentary assessment (EMA) prompts a participant completed. |
| ncravingspressed | The number of times a participant pressed the "I'm Craving" button. |
| ncustomtips_location | The number of times a participant entered a custom notification to receive at a specific location. |
| ncustomtips_time | The number of times a participant entered a custom notification to receive at a specific time of day. |
| nexplorecontentpages | The number of times a participant viewed "Tips", "FYIs" or "Inspirations" content pages available through the Explore submenu. |
| nfeelingdownpressed | The number of times a participant selected the "Feeling Down" button. |
| nfeelinggreatpressed | The number of times a participant selected the "I'm Great" button. |
| nlocationtags | The number of times a participant tagged a specific location. |
| nnotificationsreceived | The number of times a participant opened a scheduled notification from the app. |
| nprogresspressed | The number of times a participant pressed the "Progress" button to view their progress in their cessation journey. |
| nquitdateset | The number of times participants set a new quit date. |
| nregistrations | The number of times a participant registered their account in the app. |
| nscreensviewed | The number of screens a participant viewed within the app. |
| nslippedpressed | The number of times a participant selected the "I Slipped" button. |
| ntimetags | The number of times a participant tagged a specific time. |
| ntotalgames | The number of times a participant played a game. |
| naddlocation | The total number of times a participant entered a location at which to receive a location-based notification. |
| *Binary App Feature Use Variables (n=2)* | |
| noquitdate_bin | Did a participant opt not to select a quit date while setting up their profile (yes or no). |
| quitdatereset_bin | Did a participant reset their quit date at least once (yes or no). |
| *Other Variables (n=2)* | |
| phonetype | Whether a participant had an iPhone or an Android. |
| studyarm | Whether a participant had been assigned to the incentivized EMA arm or the non-incentivized EMA arm. |

**Table S3.** Sensitivity analyses: logistic regression models predicting short-term cessation from participant characteristics only (model 1) or participant characteristics and predicted probabilities of cessation from SML model including count app feature use variables (model 2).

|  | | *Model 1* | | *Model 2* | |
| --- | --- | --- | --- | --- | --- |
| Variable | | Odds Ratio | 95% CI | Odds Ratio | 95% CI |
| (Intercept) | | 1.73 | [0.00, 1920.03] | 1.16 | [0.00, 1883.37] |
| White Non-Hispanic | | 0.26 | [0.02, 3.00] | 0.26 | [0.02, 3.05] |
| Female | | 0.92 | [0.07, 12.54] | 1.26 | [0.07, 21.58] |
| Education | |  |  |  |  |
|  | More than high school degree | 0.68 | [0.03, 14.04] | 0.65 | [0.03, 15.04] |
| PHQ-9 score | | 1.01 | [.85, 1.19] | 1.05 | [0.85, 1.29] |
| Sexual minority status | | 0.30 | [0.03, 3.23] | 0.33 | [0.03, 3.93] |
| Age | | 1.01 | [0.93, 1.10] | 1.01 | [0.92, 1.10] |
| Fagerstrom Score | | 0.82 | [0.51, 1.33] | 0.81 | [0.50, 1.32] |
| Quit attempt in past year | | 1.98 | [0.10, 19.62] | 1.43 | [0.05, 40.45] |
| Polyuse | | 2.42 | [0.30, 19.62] | 2.53 | [0.29, 22.13] |
| SML-predicted probabilities | |  |  | 3.07 | [0.15, 61.33] |
| Observations (N) | | 30 | | 30 | |
| Pseudo R^2^ | | 0.14 | | 0.15 | |
| **p*< .05; ***p*<.01 | | | | | |


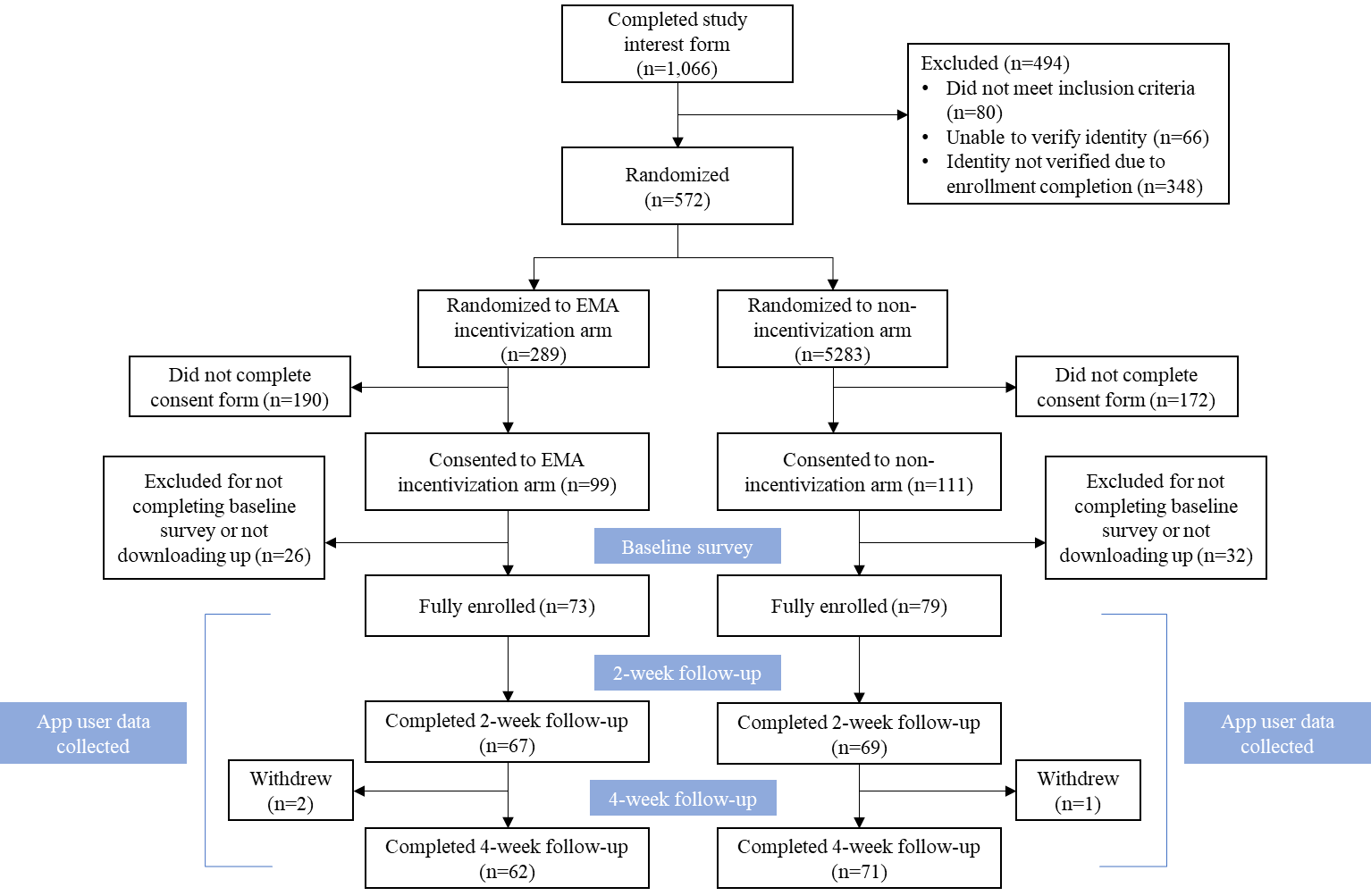


**Figure S1.** Participant recruitment, sample allocation, and data collection in the quitSTART EMA incentivization trial.


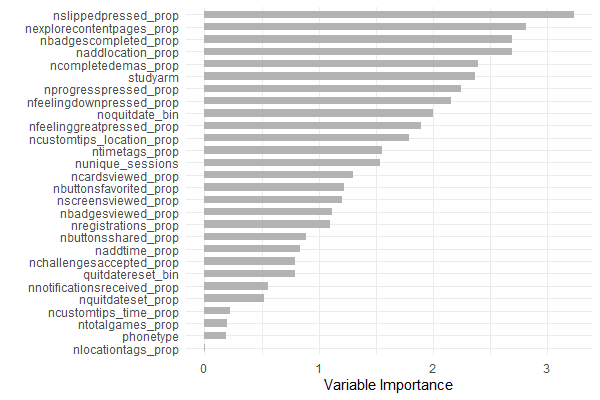


**Figure S2.** Variable importance of all variables (n=28) included in main supervised machine learning model.


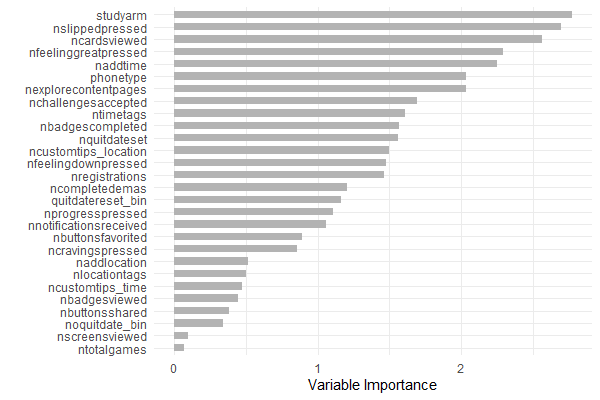


**Figure S3.** Variable Importance of All Variables (n=28) in Supervised Machine Learning Model that Included Count App Feature Use Variables
